# Current topics in liver surgery

**DOI:** 10.1002/ags3.12233

**Published:** 2019-02-15

**Authors:** Shintaro Yamazaki, Tadatoshi Takayama

**Affiliations:** ^1^ Department of Digestive Surgery Nihon University School of Medicine Tokyo Japan

**Keywords:** adverse event, mortality, surgical criteria, surgical treatment

## Abstract

Liver resection is one of the main treatment strategies for liver malignancies. Mortality and morbidity of liver surgery has improved significantly with progress in selection criteria, development of operative procedures and improvements in perioperative management. Safe liver resection has thus become more available worldwide. We have identified four current topics related to liver resection (anatomical liver resection, laparoscopic liver resection, staged liver resection and chemotherapy‐induced liver injury). The balance between treatment effect and patient safety needs to be considered when planning liver resection. Progress in this area has been rapid thanks to the efforts of many surgeons, and outcomes have improved significantly as a result. These topics remain to be solved and more robust evidence is needed. Precise selection of the optimal procedure and risk evaluation should be standardized with further development of each topic. The present article reviews these four current topics with a focus on safety and efficacy in recent series.

## 1 INTRODUCTION

Anatomical liver resection (AR) is believed to reduce the risk of intrahepatic metastases and recurrences attributable to the invasion of tumor cells in the nearby portal veins.^1^, ^2^, ^3^, ^4^, ^5^ Some studies have reported the benefits of AR compared with non‐anatomical liver resection (NAR),^6^, ^7^, ^8^, ^9^, ^10^, ^11^ but other research has failed to confirm the same results.^12^, ^13^, ^14^, ^15^ Which category of patients is most effectively treated by AR thus remains controversial.

Laparoscopic liver resection became widespread in the 1990s and is now in common use. At first, this surgery was considered controversial, but constant improvements have been made in the procedure, techniques and surrounding materials such as energy devices, forceps and scopes. As a result, laparoscopic liver resection is now one of the standard options for liver malignancies, showing merits in the operation fields and degree of invasiveness. Recently, laparoscopic liver resection has shown superiority in terms of lower intraoperative blood loss, shorter length of hospital stay and same overall and disease‐free survival compared to open liver resection.^16^, ^17^, ^18^, ^19^, ^20^, ^21^, ^22^ However, the underlying pathologies are heterogeneous, and previous studies have included small numbers of participants and differing complication rates.^23^, ^24^, ^25^, ^26^, ^27^ Recently, results from the first randomized controlled trial (RCT) and some large cohort studies have become available.^28^ Thus, more robust evidence with which to carry out laparoscopic liver resection as a standard treatment is now available.

Over the last two decades, patients with colorectal liver metastases (CRM) have shown marked improvements in long‐term survival thanks to advances in chemotherapy and surgical techniques.^29^ However, the use of several cytotoxic agents has been associated with specific liver injuries.^30^, ^31^, ^32^, ^33^ A deeper understanding of the mechanisms of action and side‐effects of common agents is needed to achieve maximal oncological benefit while reducing adverse effects from CRM.

Associating liver partition and portal vein ligation for staged hepatectomy (ALPPS) is a novel procedure to maximize remnant liver volume to carry out extended right liver resection such as right trisegmentectomy.^34^ However, according to the international ALPPS registry, more than 15% of ALPPS were done in patients who may have had no indications for two‐stage hepatectomy.^35^ They cautioned against overuse of ALPPS and mentioned that the indications should be carefully considered. The indications for ALPPS should thus be reconsidered to balance safety and efficacy. To overcome the high morbidity after ALPPS, a modified procedure is now available.^36^, ^37^, ^38^, ^39^

## 2 ANATOMICAL LIVER RESECTION IN HEPATOCELLULAR CARCINOMA

The concept of AR was proposed in the 1930s as a right or left hepatectomy.^40^ Thereafter, in 1985, Makuuchi described ultrasonically anatomical subsegmentectomy for hepatocellular carcinoma (HCC) in which every Couinaud's segment could be completely removed.^1^ The 5‐year survival rate was better in the AR group (35%) than in the enucleation group (66%, *P* < 0.05). As a result, AR was considered theoretically effective for avoiding intrahepatic metastasis of cancer cells through the portal vein, with a preference for eradicating portal venous tumor extension in HCC. In contrast, AR requires the sacrifice of a large amount of liver parenchyma to guarantee eradication of potential vascular invasion and tumor spread through the portal vein. Some authors have described AR as too complex and offering no contribution to survival.^41^, ^42^, ^43^ Most previous studies have shown no clear evidence regarding the superiority of AR and some meta‐analyses have also reported conflicting results.^12^, ^13^, ^14^, ^15^

The current series represents a review of AR between 2001 and 2015 (Table 1). We identified 18 studies on the surgical treatment of single lesions <5 cm in diameter. Most of these papers (13 studies) were retrospective,^2^, ^3^, ^4^, ^5^, ^6^, ^7^, ^8^, ^9^, ^10^ with four matched cohorts^13^, ^41^, ^42^, ^43^ and one national survey from Japan.^12^ Sufficient number of participants was included in each study. Morbidity rate ranged from 8% to 46% with AR and from 4.8% to 42% with NAR. No obvious difference in morbidity was seen between the two procedures. At the same time, mortality associated with liver resection has improved dramatically over the last two decades, implying that significant differences may not exist between procedures.^15^

**Table 1:** Anatomical versus non‐anatomical liver resection

	First author	Year	Term	Study type	Patients	Procedure	Tumor size (cm)	Single tumor (%)	Morbidity (%)	Mortality (%)	5‐y survival (%)	Reference no.
1	Yamamoto	2001	1990‐1994	Retrospective	90	AR	2.7 (±1.0)^a^	95.1	N.A.	1.1	67	14
					114	NAR	2.5 (±1.0)^a^	96.4	N.A.	2.6	55.8	
2	Hasegawa	2005	1994‐2001	Retrospective	156	AR	3.5 (1.2‐20.5)	100	N.A.	0	66	44
					54	NAR	3 (1.2‐170)	100	N.A.	0	35	
3	Capussotti	2005	1985‐2001	Retrospective	164	AR	5.1^a^	N.A.	37.2	N.A.	33.9	6
					52	NAR	4.2^a^	N.A.	38.5	N.A.	39.3	
4	Kaibori	2006	1992‐2003	Retrospective	34	AR	4.1 (±2.1)^a^	64.7	23.5	2.9	52.7	7
					213	NAR	3.3 (±2.3)^a^	72.7	25.8	1.9	46.2	
5	Wakai	2007	1990‐2004	Retrospective	95	AR	3.5 (1.2‐17.0)	100	22	2	67	8
					63	NAR	3.0 (1.0‐12.0)	100	25	6	59	
6	Yamashita	2007	1985‐2004	Retrospective	201	AR	2.9 (±0.1)^a^	100	8	N.A.	76	9
					120	NAR	2.4 (±0.1)^a^	100	10.8	N.A.	74	
7	Cho	2007	1998‐2001	Retrospective	99	AR	3.5^a^	100	12.1	0.7	65.7	10
					69	NAR	3.1^a^	100	20.3	0.8	49.3	
8	Eguchi	2008	1994‐2001	National survey	2267	AR	3.1	100	N.A.	0.71	65.5	12
					3514	NAR	2.8	100	N.A.	0.86	62.4	
9	Kobayashi	2008	1990‐2004	Retrospective	106	AR	3.0 (1.1‐14.0)	100	17	0	54	11
					127	NAR	2.8 (1.0‐14.5)	100	21	0	61	
10	Dahiya	2010	1983‐2002	Retrospective	159	AR	N.A.	N.A.	46	1.8	47.5	45
					214	NAR	N.A.	N.A.	42	0	49.4	
11	Kamiyama	2010	1990‐2006	Retrospective	169	AR	3.1^a^	100	N.A.	N.A.	83	2
					153	NAR	2.6^a^	100	N.A.	N.A.	65.3	
12	Kang	2010	1998‐2005	Retrospective	146	AR	2.8 (±0.8)^a^	100	17.8	N.A.	48	3
					21	NAR	2.7 (±0.9)^a^	100	4.8	N.A.	40	
13	Yamazaki	2010	1994‐2007	Retrospective	111	AR	3.1^a^	100	46	1.8	47.5	4
					98	NAR	2.7^a^	100	42	0	49.4	
14	Kudo	2014	2000‐2012	Retrospective	121	AR	3.3^a^	96	N.A.	0.4 (total)	63	5
					112	NAR	2.6^a^	96	N.A.		69	
15	Okamura	2014	2002‐2013	Matched cohort	64	AR	3.0 (7.0‐16.0)	100	21.9	0	71	41
					64	NAR	2.5 (1.0‐16.0)	100	18.8	0	79.7	
16	Cucchetti	2014	2001‐2010	Matched cohort	149	AR	3.0 (2.0‐4.0)	N.A.	N.A.	N.A.	65.8	42
					149	NAR	3.0 (2.0‐4.1)	N.A.	N.A.	N.A.	52.9	
17	Marubashi	2015	1981‐2012	Matched cohort	329	AR	3.5 (±2.0)^a^	78.4	N.A.	N.A.	53.9 (2 y)	43
					329	NAR	3.4 (±2.2)^a^	74.5	N.A.	N.A.	53.8 (2 y)	
18	Hirokawa	2015	2001‐2005	Matched cohort	72	AR	3.0 (0.5‐5.0)	100	24	0	79	13
					72	NAR	3.0 (1.0‐5.0)	100	10	0	84	

Data are expressed as median (range).

AR, anatomical liver resection; N.A., not applicable; NAR, non‐anatomical liver resection.

a Mean.

The survival benefit of AR remains controversial. In retrospective studies, 5‐year overall survival has tended to be better with anatomical resection.^2^, ^6^, ^7^, ^10^, ^14^, ^44^ However, a large cohort in a national survey from Japan demonstrated that AR showed superiority in neither overall survival nor disease‐free survival compared to NAR.^12^ In subgroup analysis, superiority of AR was found only for tumors between 2 and 5 cm in diameter. Matched cohort studies showed that some populations (absence of vascular invasion, tumor diameter >2.0 cm, degree of differentiation) were associated with better 5‐year overall survival following AR.^42^ Meta‐analysis of both 5‐year disease‐free survival and 5‐year overall survival has shown significantly better results with AR than with NAR.^15^ This result makes sense, in that HCC <2 cm are generally effectively treated with other treatment options such as radiofrequency ablation. In cases with tumors larger than 5 cm, the high frequency of vascular invasion may impede the local treatment effects of AR. AR thus appears to have limited beneficial effects on survival in all patients. Results from further clinical studies such as large RCT are awaited to clarify which categories are suitable for AR.

## 3 LAPAROSCOPIC LIVER RESECTION

Use of laparoscopic surgery for digestive procedures has increased rapidly and the approach is now mainstream in this area. Laparoscopic surgery for hepatobiliary‐pancreatic surgery has also spread quickly over the last decade, with the optimization of procedures and good selection criteria depending on tumor location contributing to improved effectiveness and safety.^46^, ^47^, ^48^ The merits of laparoscopic surgery in liver resection are thought to be the magnified view and reduced invasiveness of the operation.^49^ Compared to open liver resection, in recent reports, less blood loss from the hepatic veins during liver transection contributes to better surgical outcomes with laparoscopic surgery.^50^, ^51^, ^52^

The history of laparoscopic liver resection is relatively long, but marked differences can be seen in procedures, patient populations and outcomes between the most recent decade and previous years. We therefore reviewed a total of 23 recent reports concerning laparoscopic liver resection from 2008, comprising 13 matched cohort studies^16^, ^17^, ^18^, ^19^, ^20^, ^21^, ^22^, ^23^ and nine retrospective studies^24^, ^25^, ^26^, ^27^, ^58^, ^59^, ^60^, ^61^ and one RCT (Table 2).^28^

**Table 2:** Laparoscopic liver resection

	First author	Year	Term	Study type	Patients	Disease	Procedure	Tumor size (cm)	Op. time (min)	Blood loss (mL)	Morbidity (%)	Mortality (%)	5‐y survival (%)	Reference no.
1	Topal	2008	2002‐2007	Matched cohort	76	N.A.	OLR	N.A.	N.A.	300 (5‐4000)	28.9	N.A.	N.A.	16
					76		LLR	N.A.	N.A.	150 (10‐7000)	7.9	N.A.	N.A.	
2	Tsinberg	2009	2006‐2008	Retrospective	43	Mixed	OLR	4.2 (±0.3)^a^	172 (±12)^a^	299.6 (±33.6)	16	0	N.A.	24
					31		LLR	3.9 (±2.7)^a^	201 (±15)^a^	122.5 (±45.4)	13	0	N.A.	
3	Castaing	2009	1997‐2007	Matched cohort	60	CRM	OLR	4.0 (8.0‐16.0)	N.A.	N.A.	N.A.	N.A.	45	17
					60		LLR	3.0 (5.0‐8.0)	N.A.	N.A.	N.A.	N.A.	74	
4	Dagher	2009	1998‐2002	Matched cohort	50	Mixed	OLR	4.9 (±3.2)^a^	328 (±10.6)^a^	735.2 (±74.4)	34	2	N.A.	23
					22		LLR	4.3 (±7.6)^a^	360 (±20.3)^a^	519.5 (±93.4)	9	0	N.A.	
5	Sarpel	2009	1997‐2007	Matched cohort	56	HCC	OLR	4.3^a^	N.A.	N.A.	N.A.	N.A.	N.A.	53
					20		LLR	4.3^a^	N.A.	N.A.	N.A.	N.A.	N.A.	
6	Ito	2009	1998‐2008	Matched cohort	65	Mixed	OLR	3.4 (0.9‐13.0)	138^a^	200^a^	43	N.A.	56.2 (3 y)	18
					65		LLR	3.3 (0.4‐14.4)	170^a^	100^a^	14	N.A.	72.3 (3 y)	
7	Tranchart	2010	1999‐2008	Matched cohort	42	HCC	OLR	3.7^a^	221^a^	723.7^a^	40.4	2.4	37.2	19
					42		LLR	3.6^a^	233^a^	364.3^a^	21.4	2.4	45.6	
8	Vanounou	2010	2002‐2008	Retrospective	29	Mixed	OLR	4.1 (±3.6)	249^a^	N.A.	24	0	N.A.	25
					44		LLR	5.1 (±2.9)	245^a^	N.A.	16	0	N.A.	
9	Cannon	2012	1995‐2010	Matched cohort	35	CRM	OLR	4 (±2)^a^	N.A.	392 (±324)^a^	49	0.7	37	20
					35		LLR	4 (±3)^a^	N.A.	202 (±180)^a^	23	0	36	
10	Johnson	2012	2004‐2011	Retrospective	124	Mixed	OLR	5.72^a^	234^a^	833 (±1008)^a^	10.4	0.8	N.A.	58
					88		LLR	5.37^a^	238^a^	697 (±739)^a^	6.8	1.1	N.A.	
11	Bhojani	2012	2006‐2010	Retrospective	114	Mixed	OLR	3.6 (0.8‐16.7)	270 (137‐500)	250^a^	25	0	N.A.	59
					57		LLR	4.5 (0.9‐19.0)	240 (128‐605)	500^a^	39	2	N.A.	
12	Slim	2012	2008‐2011	Matched cohort	46	Mixed	OLR	4.3 (1.2‐9)	170 (85‐315)	200 (50‐2000)	39.1	2.2	N.A.	54
					46		LLR	3.2 (1.3‐8.3)	155 (45‐400)	100 (10‐800)	17.4	0	N.A.	
13	Gustafson	2012	2006‐2009	Retrospective	49	Mixed	OLR	5.1^a^	N.A.	N.A.	48.8	4.1	89.8 (1 y)	26
					27		LLR	2.6^a^	N.A.	N.A.	22.2	0	85.2 (1 y)	
14	Kobayashi	2013	1997‐2011	Retrospective	27	HCC	OLR	2.0^a^	185 (120‐430)	450 (50‐2200)	0	0	62 (3 y)	27
					24		LLR	2.2^a^	198 (45‐394)	110 (0‐1180)	0	0	50 (3 y)	
15	Medbery	2014	2008‐2012	Matched cohort	57	Mixed	OLR	8	222	737	44	4	N.A.	55
					48		LLR	5.9	175	214	28	5	N.A.	
16	Komatsu	2016	2006‐2014	Matched cohort	38	HCC	OLR	9.2	295	113	61	0	77.2	21
					38		LLR	6.7	371	190	32	0	85.4	
17	Ratti	2015	2011‐2015	Matched cohort	147	Mixed	OLR	6	200	268	26	0	N.A.	56
					49		LLR	5	259	208	22	2	N.A.	
18	Nomi	2015	1998‐2014	Retrospective	28	Mixed	OLR	N.A.	273	423	57	4	N.A.	60
					183		LLR	N.A.	279	465	55	3	N.A.	
19	Yoon	2017	2008‐2015	Matched cohort	115	HCC	OLR	5.8^a^	202	136^a^	17	0	100 (2 y)	57
					37		LLR	3.1^a^	33	125^a^	5	0	88.8 (2 y)	
20	Cheung	2016	2004‐2014	Matched cohort	330	HCC	OLR	2.9 (0.8‐10.0)	255 (45‐912)	410 (20‐5000)	24.4	N.A.	67.4	22
					110		LLR	2.6 (0.6‐10.0)	185 (50‐756)	150 (10‐1500)	10	N.A.	83.7	
21	Xiang	2016	2012‐2015	Retrospective	207	HCC	OLR	6.9 (±1.5)^a^	236 (117‐466)	456 (50‐2000)	35.7	1	82.2 (3 y)	61
					128		LLR	6.7 (±1.5)^a^	234 (105‐501)	481 (80‐3000)	20.3	0.8	81.4 (3 y)	
22	Li	2017	2005‐2010	Retrospective	87	HCC	OLR	2.3 (±0.5)^a^	140 (±52.9)^a^	85 (10‐275)	73.6	N.A.	72.4	62
					133		LLR	2.0 (±0.5)^a^	129 (±41.8)^a^	79 (20‐200)	42.9	N.A.	77.4	
23	Fretland	2018	2012‐2016	RCT	144	CRM	OLR	N.A.	120 (106‐134)	200 (126‐273)	31	1	N.A.	28
					129		LLR	N.A.	123 (108‐138)	300 (224‐375)	19	0	N.A.	

Data are expressed as median (range).

CRM, colorectal metastasis; HCC, hepatocellular carcinoma; LLR, laparoscopic liver resection; OLR, open liver resection.

Mean.

The general indications for laparoscopic resection in each study were lesions <5 cm in diameter or systematic lobectomy. Among these, the consensus was reached that operation time was significantly longer, but blood loss was lower with laparoscopic surgery than with open liver resection.^18^, ^23^, ^24^, ^54^ Morbidity appears to be better with laparoscopic liver resection, attributable to the lower rate of infectious subcutaneous complications following reductions in the length of the skin incision.^16^, ^18^, ^19^, ^20^, ^26^, ^54^ The mortality rate with laparoscopic liver resection has now decreased and optimal safety is ensured in high‐volume centers. Among the studies examined in this report, some authors noted that survival benefits did not differ between laparoscopic and open liver resection.^19^, ^20^, ^26^, ^27^

Recently, the results of a multi‐institutional large cohort meta‐analysis have become available.^63^ The mortality rate was only 0.4% (37 of 9527 patients), comparable to that in the Japanese national survey of open liver resection (0.4%‐0.5%).^12^ In cases of minor resection, mean intraoperative blood loss was 322 mL with laparoscopic liver resection, and 572 mL with open conventional liver resection, whereas values of 619 and 1299 mL, respectively, were seen with major liver resection. The morbidity rate was significantly better with laparoscopic liver resection than with open liver resection for both minor (13.5% vs 30.5%) or major liver resection (22.4% vs 45.6%).^63^ However, blood loss with open liver resection in these studies seemed extraordinarily high in high‐volume hepatobiliary centers.^64^, ^65^

Development of the laparoscopic surgery procedure shows non‐inferiority even when HCC was in an unfavorable location.^66^ This Italian multicenter study mentioned that even with HCC located on the posterior segment, the procedure can be safely carried out with a conversion rate of 17.8%. In contrast, conversion as a result of unfavorable intraoperative events resulted in worse outcomes during laparoscopic liver resection.^67^ In tertiary referral centers, the conversion rate in laparoscopic surgery was 10% among 1184 major resections in 1996‐2014 and 7.8% among 2861. A history of neoadjuvant chemotherapy, previous liver resection, extent of resection and difficult location are independent predictors of the need for conversion.

Recently, results from the RCT to compare laparoscopic and open liver resection for CRM have become available.^28^ In brief, median complexity score and tumor distribution were not statistically significant, but most of the participants in this study had fewer than two tumors and median resection volume was <100 g. Complication rate and postoperative hospital stay were significantly better in laparoscopic liver resection. Differing from the previous studies,^19^, ^24^, ^27^, ^55^ operation time was not significantly longer but intraoperative blood loss was less with laparoscopic liver resection. These results suggest that laparoscopic liver resection is catching up with conventional open liver surgery in many regards and shows superiority when carried out for optimal tumor conditions. Future prospective studies will show which conditions are more favorable for laparoscopic and conventional open liver resection.

## 4 ASSOCIATED LIVER PARTITION AND PORTAL VEIN LIGATION FOR STAGED HEPATECTOMY

Insufficient volume after liver resection is one of the independent predictors for postoperative liver failure and is closely related to high mortality.^68^ The current consensus is that more than 30% of normal liver parenchyma or more than 40% of diseased liver parenchyma should be preserved when planning operations to secure patient safety. Thus, liver functional reserve and parenchyma volume should be precisely evaluated.

When the volume of remnant liver parenchyma is small, portal vein embolization (PVE) before liver resection is recommended to increase the remnant liver parenchyma.^69^ This procedure is usually adopted in right liver resection and results in an approximately 10% increase in the volume of remnant liver.^70^, ^71^ Lower limits for an indication of PVE are approximately 30% of the remnant parenchymal volume in normal liver and 40% in chronic liver disease.^72^ PVE is now widely accepted as a useful option for certain patients who may require extensive hepatectomy. When the estimated remnant liver volume after liver resection is approximately 30% of the total liver volume, ALPPS is planned to increase the volume of remnant liver.^34^ ALPPS involves simultaneous liver partition and portal vein ligation prior to the liver resection. ALPPS provides great regeneration within the short term, but indications for this procedure are now controversial because of high mortality and morbidity.

We reviewed the current series concerning ALPPS from 2012. There were 18 retrospective studies,^34^, ^73^, ^74^, ^75^, ^76^, ^77^, ^78^, ^79^ including three multicenter studies^76^, ^83^, ^84^ (Table 3). However, the number of participants in each study was small. We compared the current results of the five PVE papers in the same term as references.^85^, ^86^, ^87^, ^88^, ^89^ The number of patients in PVE groups was sufficient to assess the outcomes. The most common current indication for ALPPS was multiple CRM, which suggests that most patients have good liver functional reserve. In contrast, the common indications for PVE were Klatskin tumor, HCC and colorectal metastasis. Rates of increase in liver volume were surprisingly different. With the ALPPS procedure, median rate of increase in remnant liver parenchyma over the first 2 weeks ranged from 48% to 113.1%, compared to 7.4% to 12% with PVE.

**Table 3:** Associated liver partition and portal vein ligation for staged hepatectomy (ALPPS) and portal vein embolization (PVE)

	First author	Year	Term	No. of patients	Procedure	Study type	Disease (%)	Increased liver volume (%)	Blood loss in liver partition (mL)	Time from treatment to assessment (days)	Morbidity (%)	Mortality (%)	Reference no.
1	Schnitzbauer	2012	2007‐2011	25	ALLPS	Retrospective	CRM (56)	74 (21 to 192)	320 (150‐7500)	9	64	12	34
2	Torres	2013	2011‐2012	39	ALLPS	Retrospective	CRM (82)	83 (47 to 212)	N.A.	14	59	12.8	73
3	Nadalin	2014	2010‐2013	15	ALLPS	Retrospective	CRM (33)	87 (23.8 to 161)	N.A.	13	67	29	74
4	Robles	2014	2011‐2013	22	ALLPS	Retrospective	CRM (77.3)	61 (33 to 189)	100 (0‐900)	7	63	9	75
5	Schadde	2014	2012‐2013	202	ALLPS	Retrospective (multicenter)	CRM (58)	86^a^	N.A.	10	40	9	76
6	Kremer	2015	2011‐2014	19	ALLPS	Retrospective	CRM	74 (±35)^a^	1380 (200‐700)	8	68	16	77
7	Hernandez‐Alejandro	2015	2012‐2013	14	ALLPS	Retrospective	CRM (80.6)	93 (±28)^a^	725 (±85)^a^	8	36	0	78
8	Truant	2015	2011‐2013	62	ALLPS	Retrospective	CRM (63)	48 (−15.3 to 192)	494 (±35)^a^	8	80.6	12.9	79
9	Alvarez	2015	2011‐2014	30	ALLPS	Retrospective	CRM (64)	89.7 (21 to 287)	N.A.	6	53	6.6	80
10	Lang	2015	2007‐2014	16	ALLPS	Retrospective	CRM (56.3)	113.1 (38.6 to 207.7)	N.A.	9	64	12.5	81
11	Chan	2016	2013‐2015	17	ALLPS	Retrospective	HCC	48.7^a^	500 (100‐2000)	8	15.3	7.7	82
12	Røsok	2016	2012‐2014	36	ALLPS	Retrospective (multicenter)	CRM (69.4)	67 (−17 to 238)	675 (150‐5600)	6	92	0	83
13	Serenari	2016	2012‐2014	50	ALLPS	Retrospective (multicenter)	CRM (44)	N.A.	N.A.	N.A.	54	20	84
14	Sakuhara	2012	1999‐2009	143	PVE	Retrospective	Klatskin (47.6)	10.7 (±6.7)^a^	–	17	6.3	0	85
15	Leung	2014	1999‐2012	153	PVE	Retrospective	CRM (89.5)	9.64 (6.75 to 12.36)	–	27	56.8	1.3	86
16	Shindoh	2014	1995‐2012	358	PVE	Retrospective	CRM (60.6)	N.A.	–	32	25.8	3.8	87
17	Sofue	2014	2007‐2011	83	PVE	Retrospective	Klatzkin (44.6)	12 (5 to 8)^a^	–	17	5	0	88
18	Cazejust	2015	2009‐2013	63	PVE	Retrospective	HCC (63.3)	11 (±7)^a^	–	24	11.1	N.A.	89

Data are expressed as median (range).

CRM, colorectal metastasis; HCC, hepatocellular carcinoma.

a Mean.

Transfusion rate (%).

The most concerning problems in ALPPS are morbidity and mortality. Morbidity rate in ALPPS ranged from 15.3% to 92%, and mortality rate ranged from 0% to 29%. Severe complications appeared to be frequent after this procedure. In the first ALPPS procedures, intraoperative blood loss to partition the liver parenchyma was too high, ranging from 100 to 725 mL. Meta‐analysis confirmed the results of operation‐related outcomes.^90^, ^91^, ^92^ This procedure is therefore considered to be under development and modifications of the procedure and revision of its indications will be required in order to improve safety.

To obtain safe procedures, some modifications to the original ALPPS have been advocated including an anterior approach with complete parenchymal division down to the IVC, an in situ split using an anterior approach followed by PVE by interventional radiology, and partial transection using the anterior approach.^36^, ^37^, ^38^, ^39^, ^93^ These modified techniques have contributed to decreases in the mortality and morbidity of ALPPS. Recently, an analysis of the international ALPPS registry cautioned against overuse of ALPPS.^35^ One‐third of ALPPS procedures for CRM were carried out without objective indications. Thus, keeping to strict indications for ALPPS would mean the procedure is done only when tri‐segmentectomy is needed for the purposes of addressing a wide tumor distribution.

## 5 CHEMOTHERAPY‐ASSOCIATED LIVER DAMAGE

Systemic chemotherapy has no doubt changed the current strategy for patients with advanced CRM. The prognosis has improved significantly on the basis of current combination chemotherapies (5‐fluorouracil, and oxaliplatin or irinotecan) with humanized monoclonal antibodies. As a result, liver resection provides potential “cure” in 10%‐30% of patients with initially unresectable CRM.^29^ However, giving long‐term chemotherapy induces chronic liver damage and is considered harmful for the prospects of future liver resection. Oxaliplatin‐induced liver damage is termed “blue liver,” pathologically appearing as sinusoidal obstruction^29^, ^30^, ^31^, ^94^ and nodular regenerative hyperplasia in the liver parenchyma.^33^ Irinotecan‐induced liver damage is called “yellow liver,” representing steatosis or steatohepatitis in liver parenchyma.^94^, ^95^ The parenchymal damage from chemotherapy negatively influences the postoperative outcomes. However, the pathophysiological background to such damage remains insufficiently understood.

### 5.1 Sinusoidal injury

Sinusoidal obstruction syndrome (SOS) was first described in patients given pyrrolizidine alkaloids.^96^ The key pathological feature is sinusoidal dilatation with hepatocyte atrophy. SOS is categorized into four grades (0‐3) according to pathological changes, depending on the duration of chemotherapy. SOS progresses to perisinusoidal fibrosis and nodular regenerative hyperplasia. Oxaliplatin increases the risk of developing sinusoidal injury by approximately 2.22‐ to 4.36‐fold when patients receive more than six cycles of chemotherapy.^96^

### 5.2 Hepatic steatosis and hepatitis

Liver steatosis and steatohepatitis induced by chemotherapy are thought to be mainly induced by irinotecan regimens. Postoperative risks appear to differ between steatosis and steatohepatitis. The pathological features vary from simple steatosis to steatohepatitis, hepatic fibrosis and cirrhosis, depending on the duration of parenchymal injury. The most widely used grading system was proposed by Kleiner et al, which categorizes features into four grades depending on the degree of steatosis (<5%, 5%‐33%, 33%‐66% and >66%).^97^ Steatohepatitis progresses to hepatic fibrosis and cirrhosis. Some reports have described high‐grade steatosis as associated with a threefold increase in postoperative mortality.^98^ However, whether hepatic steatosis alone increases the risk of postoperative mortality remains controversial. In contrast, steatohepatitis is known to increase the risk of postoperative mortality when the patient receives more than 7.5 cycles of chemotherapy. Body mass index is one surrogate marker for high risk of hepatic steatohepatitis.^99^

We reviewed 11 papers to assess the relationship between postoperative morbidity and perioperative chemotherapy from 2003 (Table 4). These included 10 retrospective studies^30^, ^31^, ^32^, ^33^, ^94^, ^100^, ^101^, ^102^ and one RCT.^29^ The number of participants in each study varied. Most patients in the chemotherapy group were given systemic preoperative chemotherapy for metastasis from colorectal cancer. The data suggested that irinotecan‐based chemotherapy was closely associated with development of steatohepatitis.^105^ Presence of steatohepatitis has been shown to increase postoperative morbidity and mortality in non‐alcoholic steatohepatitis (NASH) patients.^106^ The pathological feature of chemotherapy‐induced liver damage resembles that in NASH patients. Some authors have cautioned that long‐term chemotherapy‐induced steatohepatitis is thus a risk for postoperative mortality. However, the optimal method for estimating liver functional reserve and how far liver resection can be safely carried out remains unconfirmed. Interruption of chemotherapy is reported to improve liver function (indocyanine green [ICG] retention rate at 15 minutes value from 17.7% to 11.6%) within 4 weeks.^107^ Therefore, estimation by ICG may be of value to estimate chemotherapy‐induced liver damage.

**Table 4:** Chemotherapy‐induced perioperative risk

	First author	Year	Term	Study type	Patients	Setting	Tumor diameter (cm)	Single tumor (%)	Blood loss (mL)	Morbidity (%)	Mortality (%)	DFS	5SU	Reference no.
1	Parikh	2003	1997‐2002	Retrospective	34	Preoperative chemo (CPT‐based)	2.0 (0.8‐6.5)	60	500^a^	29	0	N.A.	N.A.	33
					47	Surgery alone	4.3 (0.9‐10.3)	27	425^a^	49	0	N.A.	N.A.	
2	Fernandez	2005	2001‐2003	Retrospective	14	Preoperative chemo (OX‐ or CPT‐based) + surgery	N.A.	N.A.	N.A.	N.A.	0	N.A.	N.A.	94
					14	Surgery alone	N.A.	N.A.	N.A.	N.A.	0	N.A.	N.A.	
3	Karoui	2006	1998‐2002	Retrospective	45	Preoperative chemo + major surgery	3.0 (±1.7)^a^	N.A.	44.5^b^	37.8	0	N.A.	N.A.	100
					22	Major surgery	2.6 (±2.3)^a^	N.A.	45.5^b^	13.6	0	N.A.	N.A.	
4	Sahajpal	2007	2001‐2003	Retrospective	53	Preoperative chemo + surgery	4.5	N.A.	1242^a^	39.6	0	N.A.	N.A.	101
					43	Surgery alone	5.8	N.A.	1245^a^	51.2	0	N.A.	N.A.	
5	Nordlinger	2008	2000‐2004	RCT	182	Perioperative chemo (OX‐based)	N.A.	51	N.A.	25	1	28.1 (3 y)	N.A.	29
					182	Surgery alone	N.A.	52	N.A.	16	1	36.2 (3 y)	N.A.	
6	Hubert	2010	2000‐2006	Retrospective	72	Perioperative chemo (OX‐based)	N.A.	47	225 (150‐1600)	57.3	0	N.A.	N.A.	30
					18	Surgery alone	N.A.	73	600 (150‐4700)^a^	50	3	N.A.	N.A.	
7	Kishi	2010	1999‐2007	Retrospective	157	Preoperative short‐term chemo + surgery	2.1 (0.4‐14.0)	36	230 (10‐1500)	3.8 (liver insufficiency)	N.A.	N.A.	N.A.	102
					62	Preoperative chemo long‐term chemo + surgery	2.7 (0.4‐10.5)	31	200 (20‐3100)	11.3 (liver insufficiency)	N.A.	N.A.	N.A.	
8	Soubrane	2010	1998‐2007	Retrospective	13	Preoperative chemo (SOS low‐grade) + surgery	6.3 (total length)^a^	N.A.	483 (±328)^a^	23.1	0	N.A.	N.A.	1
					38	Preoperative chemo (SOS high‐grade) + surgery	7.9 (total length)^a^	N.A.	880 (±960)^a^	26.3	5.3	N.A.	N.A.	
9	Pessaux	2010	200‐2009	Retrospective	26	Preoperative chemo (Cmab) + surgery	4.8^a^	88.5	1019 (±1597)^a^	34.6	0	N.A.	N.A.	103
					26	Preoperative chemo (without Cmab) + surgery	4.3^a^	88.5	708 (±452)^a^	30.8	0	N.A.	N.A.	
10	Makowiec	2011	2001‐207	Retrospective	68	Preoperative chemo + surgery	N.A.	N.A.	N.A.	50	6	N.A.	N.A.	104
					34	Surgery alone	N.A.	N.A.	N.A.	44	2	N.A.	N.A.	
11	van der Pool	2012	2003‐2008	Retrospective	53	Preoperative chemo (OX‐based) + surgery	3.5 (1‐7)	N.A.	32^b^	32.1	N.A.	32 (3 y)	N.A.	32
					51	Preoperative chemo (OX‐based + Bmab) + surgery	2.8 (1‐18)	N.A.	29^b^	25.5	N.A.	23 (3 y)	N.A.	

Data are expressed as median (range).

CPT, irinotecan; DFS, disease‐free survival; OX, oxaliplatin; RCT, randomized controlled trial; SOS, sinusoidal occlusion syndrome.

a Mean.

b Transfusion rate (%).

Whether preoperative chemotherapy increases intraoperative blood loss is a controversial issue. Intraoperative blood loss is significantly greater in pathological high‐grade SOS than in low‐grade SOS.^31^ This difference may reflect the hepatic venous pressure gradient and parenchymal stiffness, and the increasing rate of SOS is associated with morbidity after liver resection. Rate of increase in splenic volume and decrease in platelet count are predictive of SOS.^108^, ^109^, ^110^, ^111^ Reportedly, 75% of patients who received adjuvant chemotherapy had increased splenic volume and 40% of patients did not recover after discontinuation of chemotherapy.^99^, ^111^ Giving bevacizumab in combination with systemic chemotherapy reduces the occurrence of SOS.^112^ However, the duration and extent to which liver function can recover remains unclear.

## 6 CONCLUSION

Liver resection has gained wide use with more precise preoperative evaluation of hepatic functional reserve and tumor status. We now have the Japanese treatment algorithm to aid in decision‐making for the surgical treatment of HCC, backed up by robust evidence.^113^ Anatomical resection for HCC is a more complex procedure that results in greater intraoperative blood loss and longer operation time. Overall survival from HCC is multifactorial, but a significant positive impact on survival has been observed in anatomical resection. However, no RCT have determined whether anatomical resection is an essential technique for HCC. The results of our ongoing RCT might allow the establishment of optimized procedures.

There is currently no doubt that laparoscopic liver surgery offers comparable mortality to open liver resection when the optimal location and procedure are selected.^22^, ^28^, ^57^, ^61^, ^62^ Laparoscopic liver resection is technically demanding and requires technical command of both liver and laparoscopic surgery. Recently, consensus guidelines to determine suitable tumor location and optimal procedure for laparoscopic liver resection have been developed. A pre‐registration system has also been available since 2015 to ensure safety and to maintain the quality of laparoscopic liver resection.^46^, ^114^, ^115^

High morbidity and mortality rates remain the most critical problem in ALPPS, which is now a widely accepted option for “marginal resectable” patients with Klatskin tumor or multiple CRM. However, we must keep in mind that patients with primary unresectable CRM experience a high rate of recurrence and receive long‐term chemotherapy. Thus, careful evaluation for chemotherapy‐induced liver injury is needed to maintain quality for ALPPS. Use of a risk score for ALPPS and further refinement of the indications for ALPPS are warranted.^35^, ^116^ To overcome these problems, a pre‐registration system is now available and may provide a way to obtain safer indications for ALPPS.^117^

Current chemotherapies are obviously powerful, but are closely associated with liver injury, which is regimen‐specific.^118^ We should keep in mind that preoperative long‐term chemotherapy is a risk factor for liver injury. Thus, in cases of major liver resection in patients who have received long‐term chemotherapy, precise evaluation of liver functional reserve and volume should be carried out.

We have presented topics on the current treatment strategies for liver cancers, showing that surgery has the power to dramatically improve prognosis and the primary option of choice. It is important to take into account the balance between tumor distribution and difficulty of operation. Consideration of the advantages of techniques and treatment effects is important and the method of the operation should not be the purpose of the treatment. Safety is the first priority in surgical treatment and should be refined using high‐level evidence as it becomes available.
